# Tumors of the Face

**Published:** 1849-07

**Authors:** 


Tumors of the Face.—It is well known to those who have had experience in surgical matters, that morbid growths, or new tormations in any part of the body, cannot be got rid of by the internal use of deobstruent remedies, as they are called, nor yet by local applications, however potent and stimulating; in fact, much mischief often arises from meddlesome and imprudent interference with tumors of various kinds; their growth is often hastened, and their connections with their coverings and with neighboring parts, is often rendered more intimate. You now and then, however, see glandular swellings give way under constitutional, assisted by local treatment. Occasionally tumors occur in the face, occupying a space below the zygomatic process, anterior to the masseter muscle, and also under the lower jaw. Swellings in these situations are very likely to depend on inflammation and irritation in the mouth. They are sometimes firmly fixed, involve a considerable portion of the face and neck, and project outwardly to a great extent; but on the cause being removed, and the general health improved, they become gradually absorbed and disappear. These are very different from the sarcomatous tumors of this region, which are generally loosely connected to the surrounding parts, and are of very slow growth. Care must be taken to ascertain the nature of the disease before proposing a severe operation.—*Liston's Lectures —Lancet.*

